# Health-related quality of life and burden of illness in adults with newly diagnosed attention-deficit/hyperactivity disorder in Sweden

**DOI:** 10.1186/s12888-018-1803-y

**Published:** 2018-07-13

**Authors:** E. Ahnemark, M. Di Schiena, A.-C. Fredman, E. Medin, J. K. Söderling, Y. Ginsberg

**Affiliations:** 1 Shire, Vasagatan 7, SE-111 20 Stockholm, Sweden; 2 Prima Child and Adult Psychiatry AB, Stockholm, Sweden; 3 PAREXEL International, Stockholm, Sweden; 4 Department of Learning, Informatics, Management and Ethics, Karolinska Institute, Stockholm, Sweden; 5 Bell Analytics, Stockholm, Sweden; 6 Department of Medical Epidemiology and Biostatistics, Karolinska Institute, Stockholm, Sweden; 7 Department of Clinical Neuroscience, Centre for Psychiatry Research, Karolinska Institute, Stockholm, Sweden; 8 Present Address: Psychiatry Centre, Stockholm County Council, Södertälje, Sweden

**Keywords:** ADHD, HRQoL, Psychiatric comorbidities

## Abstract

**Background:**

This observational, cross-sectional, retrospective chart review aimed to identify factors determining health-related quality of life (HRQoL) in adults with newly diagnosed attention-deficit/hyperactivity disorder (ADHD) in Sweden.

**Methods:**

Adult participants with a new clinical diagnosis of ADHD were enrolled from two specialist outpatient clinics in Stockholm, Sweden, from 2013 to 2015. Data extracted from patient records included demographics, clinical characteristics and comorbid psychiatric diagnoses identified using the Mini International Neuropsychiatric Interview (MINI). Depression severity was assessed using the Montgomery–Åsberg Depression Rating Scale – Self-reported (MADRS-S). The self-rated five-dimension EuroQol questionnaire (EQ-5D) was used to measure HRQoL. Predictors of EQ-5D index score were identified using multivariate linear regression adjusting for age, sex, education level, and main income source.

**Results:**

The mean age of the 189 enrolled patients was 35.2 years (standard deviation [SD], 12.3), and 107 (57%) were female. Psychiatric comorbidities were present in 92 patients (49%), with anxiety and depression being the most common diagnoses. The mean EQ-5D index score was 0.63 (SD, 0.28). Low EQ-5D index scores were significantly associated with high MADRS-S scores, multiple comorbid psychiatric disorders, low educational achievement, female sex, and not having a main income derived from employment or self-employment.

**Conclusions:**

These findings suggest that adults with newly diagnosed ADHD experience low HRQoL, which may often be exacerbated by psychiatric comorbidities such as anxiety and depression. Patients presenting with ADHD and psychiatric comorbidities in adulthood may require particular care and resources in the management of their ADHD.

**Electronic supplementary material:**

The online version of this article (10.1186/s12888-018-1803-y) contains supplementary material, which is available to authorized users.

## Background

Attention-deficit/hyperactivity disorder (ADHD) is estimated to affect between 2.5 and 5.0% of adults worldwide [1–3]. Although originally considered to be a disorder of childhood, ADHD is now recognized to persist into adulthood in approximately 65% of cases [4–7]. While many adults with newly diagnosed ADHD may have experienced symptoms as children [6], two recent studies have suggested that, in some cases, ADHD symptoms may not manifest until adulthood [8, 9]. Results from a study in Sweden demonstrated that the number of adults diagnosed with ADHD increased year on year from 2006 to 2011 [10], and in 2013 ADHD was diagnosed in 2.7% of adult psychiatric patients in Sweden [11]. Despite the increasing recognition of ADHD in the adult Swedish population, a recent registry study reported that patients with psychiatric symptoms may undergo years of treatment before receiving an ADHD diagnosis [11]. Nylander et al. found that diagnosis of ADHD in adults took, on average, 3 years from the initial point of contact with psychiatric services, although in some cases diagnoses were delayed for as long as 10 years [11]. Delays in diagnosis were particularly apparent if the signs and symptoms of ADHD were attributed to other psychiatric disorders, for example, anxiety disorders, which often co-occur with ADHD [11]. These findings highlight the need for increased recognition and understanding of ADHD as an adult psychiatric disorder. Similarly, the European Network Adult ADHD published a consensus document in 2010, with the aims of increasing awareness of ADHD in adults and improving patient care across Europe [12].

The effects of ADHD can have a significant negative impact on many aspects of adult life, from social and emotional well-being to professional development and financial security [13, 14]. Despite this, few studies have investigated the burden of disease in adult patients with ADHD. Swedish patient records and archives provide a rich source of data for investigating burden of illness in adults with newly diagnosed ADHD. The diagnostic procedure for adults comprises a clinical interview to assess the frequency and duration of ADHD symptoms, as well as a full neuropsychiatric investigation of functional impairments from both clinical and social perspectives. This retrospective chart review study utilized the five-dimension EuroQol questionnaire (EQ-5D) [15] to develop a predictive model of health-related quality of life (HRQoL) in adults with newly diagnosed ADHD, based on their ADHD symptoms, psychiatric comorbidities, and socioeconomic characteristics.

## Methods

### Study design and conduct

This study was an observational, cross-sectional, retrospective chart review of adult patients with newly diagnosed ADHD at one of two specialist neuropsychiatric outpatient clinics (Liljeholmen or Danderyd) in Stockholm, Sweden, between 2013 and 2015. The study was conducted in accordance with the ethical standards of the World Medical Association Declaration of Helsinki. The study was approved by the Regional Ethical Review Board in Stockholm and the need for consent was waived due to the retrospective nature of the study.

### Participants

The study population was identified from electronic medical records (EMRs) and archives of patients’ neuropsychiatric investigations from the two study sites. Only patients aged 18 years and above who underwent neuropsychiatric investigation and received a new and confirmed diagnosis of ADHD (International Statistical Classification of Diseases and Related Health Problems – 10th Revision, Swedish modification [ICD-10-SE] diagnosis code F90) [16] were enrolled in the study.

### Diagnostic investigations at Liljeholmen and Danderyd

Extensive diagnostic investigations were conducted at outpatient clinics for all enrolled patients. Patients were interviewed alongside any family members or companions (usually parents or spouse), and details of their medical history, developmental history, coexistent disorders, and current condition were recorded. A medical examination, including blood tests and drug screening, was also part of the diagnostic process. During the neuropsychiatric investigation, the frequency and duration of patients’ psychiatric symptoms were recorded, their functional impairments and cognitive and executive functioning were assessed, and routine diagnostic tests for psychiatric disorders were completed. Several different self-report scales, psychological tests, and structured interviews were used to provide a comprehensive neuropsychiatric evaluation and confirm the ADHD diagnosis (Table 1). ADHD-specific instruments included the Diagnostic Interview for ADHD in Adults, second edition (DIVA 2.0), and the Adult ADHD Self-Report Scale version 1.1 (ASRS-v1.1).

**Table 1:** Data extracted from electronic medical records and archives

Basic characteristics	Age, sex, type of referral, confirmed ADHD diagnosis, other psychiatric diagnoses (confirmed using MINI)
Social status	Marital status, parental status (biological), age of children, housing, education, main source of income
Patient history	Psychiatric history, previous confirmed psychiatric diagnoses, current registered somatic diagnoses
Family history	General and specific psychiatric and neuropsychiatric disorders in the family
Risk assessment	Previous suicide attempts, previous or current self-destructive, aggressive, or criminal behaviour
ADHD diagnostic interview	DIVA 2.0 (symptoms of attention deficit [A1] and hyperactivity/impulsivity [A2])
Patient-reported measures	Scores for ASRS-v.1.1 Screener and Symptom checklist and AUDIT, DUDIT, MADRS-S, and EQ-5D
Intelligence assessment	WAIS-IV score
Pharmacological treatments	Current psychiatric and somatic medications, recommended ADHD medication after neuropsychiatric investigation

*ADHD* attention-deficit/hyperactivity disorder, *ASRS-v.1.1* Adult ADHD Self-Report Scale version 1.1, *AUDIT* Alcohol Use Disorders Identification Test, *DIVA 2.0* Diagnostic Interview for ADHD in Adults, second edition, *DUDIT* Drug Use Disorders Identification Test, *EQ-5D* five-dimension EuroQol questionnaire, *MADRS-S* Montgomery–Åsberg Depression Rating Scale – Self-reported, *MINI* Mini International Neuropsychiatric Interview, *WAIS-IV* Wechsler Adult Intelligence Scale IV

### Data extraction

All patient-related information, including findings from the neuropsychiatric investigations, were accessed through EMRs and patient archives. Clinical researchers with knowledge and experience of treating patients with ADHD extracted anonymized patient data using data-gathering forms specifically designed for the study. The data-gathering form was based on the recommendations for neuropsychiatric investigations of patients with suspected ADHD described in recent guidelines (www.psykiatristod.se) and validated by a clinical expert.

The information extracted included social and clinical characteristics, patient and family histories, and details of any prescribed medication. Adult scores from the DIVA 2.0 and ASRS-v.1.1 Screener (6 items) and Symptom checklist (18 items) were also obtained (Table 1) [17].

Full details of any psychiatric diagnoses, made using the Mini International Neuropsychiatric Interview (MINI) Swedish version 6.0.0 [18], were also extracted from the databases. The MINI comprises a short structured interview in which patient symptoms and signs are assessed against diagnostic criteria given in the Diagnostic and Statistical Manual of Mental Disorders, fourth edition (DSM-IV) [19]. MINI-identified depression includes depressive episodes and recurrent depression (ICD-10-SE diagnosis codes F32–F33). MINI-identified anxiety includes phobias, other anxiety disorders and obsessive–compulsive disorder (ICD-10-SE, F40–F42). Autism was diagnosed separately as part of the neuropsychiatric investigation.

Other extracted data included patient-reported indications of drug and alcohol abuse, depression, and HRQoL (Table 1). HRQoL was assessed using the EQ-5D, and the Montgomery–Åsberg Depression Rating Scale – Self-reported (MADRS-S) screening instrument [20] was used to evaluate the presence and severity of depressive symptoms. For patients enrolled from the Liljeholmen clinic, MADRS-S scores were calculated from Patient Health Questionnaire 9 (PHQ-9) scores using a published regression equation (MADRS-S = [1.206 × PHQ-9] + 4.062) [21].

### Linear regression models

Potential predictors of HRQoL were identified using a linear regression model of EQ-5D index score, adjusting for age (18–29, 30–39, 40–49, or ≥ 50 years), sex, education (secondary/university or other), and main source of income (full- or part-time employment or other). A linear regression model with the same covariates was used to predict differences in EQ-5D index score between subgroups of patients with or without comorbid psychiatric diagnoses.

A multiple imputation model was employed to estimate values for missing data using five iterations per value. Covariates included in the imputation model were age, sex, study site, education, source of income, MINI-identified comorbid diagnoses, previously confirmed psychiatric disorders, previous suicide attempts, somatic diagnoses, current psychiatric medications, recommended ADHD medication after neuropsychiatric investigation, DIVA 2.0 scores, ASRS-v.1.1 scores, MADRS-S scores, and both EQ-5D index and visual analogue scale scores. Missing EQ-5D index scores were replaced for 27 patients (Additional file 1: Table S1). Patients aged 50 years or older and those recruited in Liljeholmen (as opposed to Danderyd) were more likely to have missing EQ-5D values. Although imputed values for these patients tended to be below the mean, the distributions of the observed and imputed EQ-5D index score data were similar. Multiple imputation was performed using SAS v9.3 software (SAS Institute, Cary, NC, USA).

### Statistical analysis

Differences between study sites in mean patient age and the ratio of male to female patients were analysed using *t*-tests and χ^2^ tests, respectively (*α* = 0.05). Data were analysed using SAS v9.3 (SAS Institute, Cary, NC, USA).

## Results

### Demographics and patient characteristics

The study enrolled 189 adult patients with newly diagnosed ADHD, of whom 57% were women (Table 2). The mean patient age was 35.2 years (standard deviation [SD], 12.3), and ages ranged from 18 to 72 years. There was no statistically significant difference in mean patient age between the two clinics, but a significantly larger proportion of female participants were enrolled from Danderyd than from Liljeholmen (*p* = 0.04; Table 2).

**Table 2:** Patient demographics and clinical characteristics

Characteristic	Danderyd (*n* = 101)	Liljeholmen (*n* = 88)	Overall (*N* = 189)
Sex, *n* (%)			
Female	61 (60)	46 (52)	107 (57)
Male	40 (40)	42 (48)	82 (43)
*p* value^a^			0.04
Age, years			
Mean (SD)	33.7 (12.4)	36.9 (12.0)	35.2 (12.3)
Median (range)	30 (18–72)	36 (18–66)	33 (18–72)
*p* value^b^			0.08
Confirmed ADHD diagnosis, *n* (%)			
Combined type^c^	57 (56)	57 (65)	114 (60)
Predominantly inattentive type^d^	37 (37)	12 (14)	49 (26)
Unspecified type^e^	7 (7)	19 (21)	26 (14)
DIVA 2.0, mean number of symptoms (SD)			
Attention deficit^f^ (A1)	7.8 (1.4)	6.9 (2.3)	7.4 (1.9)
Hyperactivity/impulsivity^g^ (A2)	5.3 (2.9)	5.7 (2.5)	5.5 (2.7)
Highest level of education, *n* (%)			
Primary school	36 (36)	33 (38)	69 (37)
Secondary school	46 (46)	28 (32)	74 (39)
University	11 (11)	13 (15)	24 (13)
Other^h^	8 (8)	14 (16)	22 (12)
Main source of income, *n* (%)			
Full- or part-time employment	32 (32)	38 (43)	70 (37)
Self-employment (full- or part-time)	9 (9)	9 (10)	18 (10)
Sickness benefit (short- or long-term)	23 (23)	19 (22)	42 (22)
Unemployment benefit	12 (12)	3 (3)	15 (8)
Other^i^	25 (25)	19 (22)	44 (23)
Type of referral, *n* (%)			
Self-initiated	27 (27)	13 (15)	40 (21)
School	1 (1)	2 (2)	3 (2)
Primary care	13 (13)	36 (41)	49 (26)
Psychiatric department	56 (55)	28 (32)	84 (44)
Other	4 (4)	7 (8)	11 (6)
Missing	0 (0)	2 (2)	2 (1)

*ADHD* attention-deficit/hyperactivity disorder, *DIVA 2.0* Diagnostic Interview for ADHD in Adults, second edition, *ICD-10-SE* International Statistical Classification of Diseases and Related Health Problems – 10th Revision, Swedish modification, *SD* standard deviation

^a^χ^2^ test was used to compare proportions of male and female patients between the two test sites

^b^*t*-test was used to compare the mean ages of patients enrolled at the two test sites

^c^ICD-10-SE F90.0 B: ADHD, combined type

^d^ICD-10-SE F90.0 C: ADHD, predominantly inattentive type

^e^ICD-10-SE F90.0 X: ADHD, unspecified ADHD

^f^*n* = 163 (Danderyd, 91; Liljeholmen, 72)

^g^*n* = 157 (Danderyd, 86; Liljeholmen, 71)

^h^‘Other’ includes occupational (7%), other (4%), and missing (1%)

^i^‘Other’ includes ‘studying/student loan’ (7%), ‘other’ (13%), and ‘missing’ (4%)

The majority of patients (114/189 [60%]) had diagnoses of ‘combined type’ ADHD, while 49/189 (26%) had ‘predominantly inattentive type’ and 26/189 (14%) had ‘unspecified type’ (which includes either ‘combined’ type or ‘predominantly inattentive’ type; Table 2). Of the 189 patients, most did not reach a level of education beyond secondary school; 13% had a university education. Fewer than half of the patients listed employment as their main source of income, with 37% in full- or part-time employment and 10% self-employed (either full- or part-time; Table 2).

### Clinical characteristics and treatment history

Data extracted from the MINIs revealed that almost half of patients (92/189 [49%]) had a psychiatric comorbidity (Table 3). Anxiety and depression were the most common, affecting 65/189 patients (34%) and 37/189 patients (20%), respectively. Both anxiety and depression were frequently registered alongside other MINI-identified diagnoses, but few patients had anxiety or depression diagnosed as a single comorbidity (25/189 [13%] and 11/189 [6%], respectively; Table 3). An autism diagnosis was recorded in 30/189 patients (16%).

**Table 3:** Patients’ psychiatric and somatic comorbidities and prescribed psychiatric medication

Comorbidities	Danderyd (*n* = 101)	Liljeholmen (*n* = 88)	Overall (*N* = 189)
Psychiatric, *n* (%)^a^			
Any	61 (60)	31 (35)	92 (49)
None	40 (40)	57 (65)	97 (51)
Anxiety and/or depression, *n* (%)			
Anxiety	43 (43)	22 (25)	65 (34)
Anxiety only^b^	16 (16)	9 (10)	25 (13)
Depression^c^	28 (28)	9 (10)	37 (20)
Depression only^b^	7 (7)	4 (5)	11 (6)
Both anxiety and depression^d^	17 (17)	4 (5)	21 (11)
Other, *n* (%)			
Any other psychiatric diagnosis	21 (21)	14 (16)	35 (19)
Dysthymic disorder	5 (5)	4 (5)	9 (5)
Substance-related disorders	6 (6)	2 (2)	8 (4)
Antisocial personality disorder	4 (4)	3 (3)	7 (4)
Eating disorder	5 (5)	1 (1)	6 (3)
Bipolar disorder	4 (4)	1 (1)	5 (3)
Post-traumatic stress disorder	3 (3)	1 (1)	4 (2)
Somatic comorbidity, *n* (%)			
Any	86 (85)	59 (67)	145 (77)
None	15 (15)	29 (33)	44 (23)
Pain	25 (25)	11 (13)	36 (19)
Gastrointestinal disorders	23 (23)	9 (10)	32 (17)
Allergy	21 (21)	6 (7)	27 (14)
Asthma	10 (10)	9 (10)	19 (10)
Joint problems	6 (6)	6 (7)	12 (6)
Thyroid disease	8 (8)	3 (3)	11 (6)
Lactose intolerance	6 (6)	1 (1)	7 (4)
Prescribed psychiatric medication, *n* (%)			
Any	61 (60)	47 (53)	108 (57)
None	40 (40)	41 (47)	81 (43)
Antidepressants	37 (37)	30 (34)	67 (35)
Hypnotics	24 (24)	14 (16)	38 (20)
Anxiolytics	18 (18)	19 (22)	37 (20)
Antipsychotics	3 (3)	2 (2)	5 (3)
Central nervous system stimulants	3 (3)	0 (0)	3 (2)

*MINI* Mini International Neuropsychiatric Interview

^a^Comorbid psychiatric diagnoses were based on MINI and did not include autism. An autism diagnosis was recorded in 30/189 patients (16%)

^b^This disorder is listed as the only registered comorbidity

^c^Includes recurrent and single-episode depression

^d^MINI-identified diagnosis of recurrent or single-episode depression and MINI-identified diagnosis of anxiety

The majority of patients (108/189 [57%]) had been prescribed pharmacological treatments for psychiatric disorders. Medications included antidepressants (67/189 [35%]), hypnotics (38/189 [20%]), anxiolytics (37/189 [20%]), and antipsychotics (5/189 [3%]; Table 3). Three patients (3/189 [2%]) had been prescribed ADHD medication (the central nervous system stimulant, osmotic-release oral system methylphenidate [OROS-MPH]) despite lacking a confirmed ADHD diagnosis. Following ADHD diagnosis, pharmacological ADHD treatment was recommended for 146/189 patients (77%). Most were prescribed OROS-MPH or other methylphenidate formulations (130/189 [69%]), while atomoxetine and lisdexamfetamine dimesylate were prescribed to 8/189 patients (4%) and 5/189 patients (3%), respectively.

Somatic comorbidities affected 145/189 patients (77%) at enrolment. The most frequently reported conditions were pain (36/189 [19%]), gastrointestinal disorders (32/189 [17%]), and allergy (27/189 [14%]; Table 3). Just over one-third of patients (69/189 [37%]) had been prescribed medication for these comorbidities.

### Patient-reported measures and HRQoL subgroup analyses

The mean ASRS-v.1.1 Symptom checklist score in the study population was 47.4 (SD, 13.0), indicating that the majority of patients regularly experienced multiple ADHD symptoms (Table 4). Depressive symptoms were also common, as indicated by the mean MADRS-S score of 19.8 (SD, 9.2). The mean EQ-5D index score was 0.63 (SD, 0.28), indicating poor HRQoL in the study population.

**Table 4:** Patient-reported measures of ADHD symptoms, depression, and HRQoL

Measure	Danderyd (*n* = 101)		Liljeholmen (*n* = 88)		Overall (*N* = 189)	
	*n*	Mean (SD)	*n*	Mean (SD)	*n*	Mean (SD)
ASRS-v1.1 score						
Screener	1	–	65	5.0 (1.0)	66	5.0 (1.0)
Symptom checklist	98	46.4 (13.0)	11	56.6 (8.7)	109	47.4 (13.0)
MADRS-S score^a^	93	20.3 (9.6)	72	19.1 (8.5)	165	19.8 (9.2)
EQ-5D						
Index score	95	0.64 (0.27)	67	0.61 (0.30)	162	0.63 (0.28)
VAS	91	58.1 (21.2)	66	56.4 (23.5)	157	57.4 (22.1)

*ADHD* attention-deficit/hyperactivity disorder, *ASRS-v1.1* Adult ADHD Self-Report Scale version 1.1, *EQ-5D* five-dimension EuroQol questionnaire, *HRQoL* health-related quality of life, *MADRS-S* Montgomery–Åsberg Depression Rating Scale – Self-reported, *PHQ-9* Patient Health Questionnaire 9, *SD* standard deviation, *VAS* visual analog scale

^a^MADRS-S scores were converted from PHQ-9 scores for 72 patients from Liljeholmen and one patient from Danderyd using a regression equation (MADRS-S = [1.206 × PHQ-9] + 4.062)

**Fig. 1:**
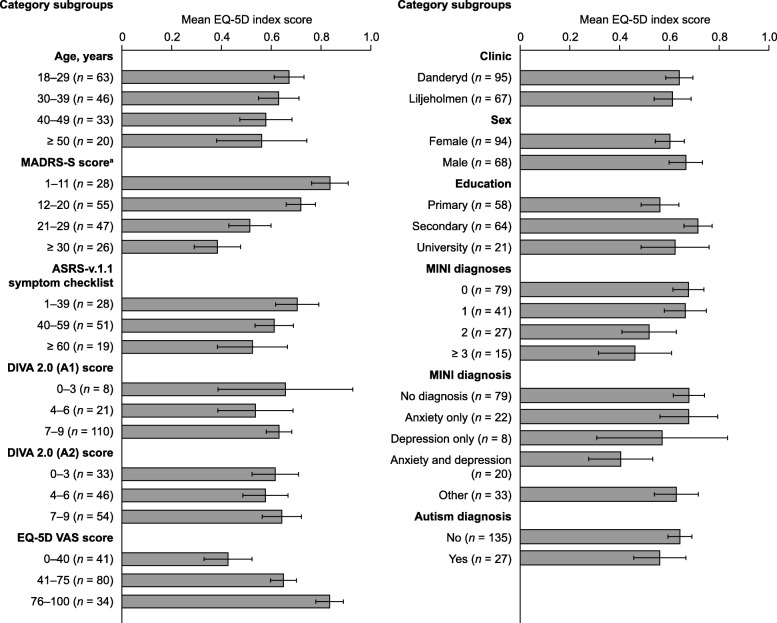
EQ-5D index score subgroup analysis**.** Error bars show 95% confidence intervals. ^a^MADRS-S derived from PHQ-9 score for patients enrolled at Liljeholmen (1.206 × PHQ-9) + 4.062. ADHD: attention-deficit/hyperactivity disorder; ASRS-v1.1: Adult ADHD Self-Report Scale version 1.1; DIVA 2.0: Diagnostic Interview for ADHD in Adults, second edition (A1: Attention deficit; A2: hyperactivity/impulsivity); EQ-5D: 5-dimension EuroQol questionnaire; MADRS-S: Montgomery–Åsberg Depression Rating Scale – Self-reported; MINI: Mini International Neuropsychiatric Interview; PHQ-9: Patient Health Questionnaire 9; VAS: visual analog scale

Subgroup analysis was used to identify trends associated with low EQ-5D index scores (indicating poor HRQoL) in the patient population (Fig. 1). Mean EQ-5D index scores tended to be lower in older patients than in younger patients, with the lowest mean index score recorded in patients aged 50 years or above (Fig. 1). Other variables associated with low EQ-5D index scores included patient-rated severe depressive symptoms (MADRS-S score ≥ 30), an ASRS-v.1.1 score of 60 or greater, and having multiple comorbid psychiatric disorders.

### HRQoL predictor analysis

**Fig. 2:**
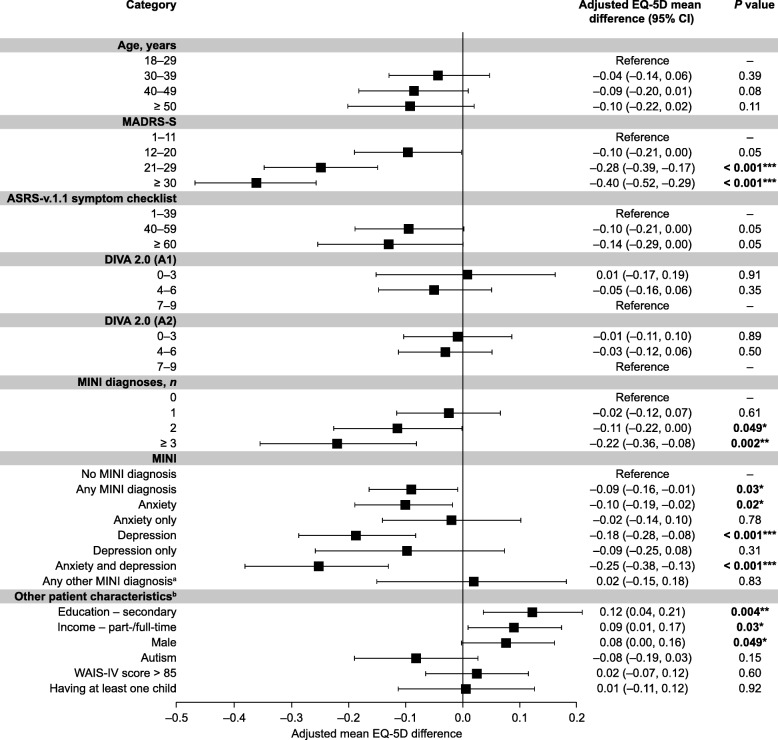
EQ-5D index score linear regression model. *P < 0.05, **P < 0.01, ***P < 0.001. Error bars show 95% confidence intervals. ^a^Any MINI diagnosis other than anxiety and depression. ^b^Reference populations for binary variables (other patient characteristics): education, secondary or university vs. no secondary; main income from full- or part-time employment vs. other sources; male sex vs. female sex; autism, confirmed diagnosis vs. no diagnosis. “Reference” indicates subgroup used for statistical comparisons with other subgroups. *p* values rounded to nearest 0.01 decimal places. ADHD: attention-deficit/hyperactivity disorder; ASRS: Adult ADHD Self-Report Scale version 1.1; CI: confidence interval; DIVA 2.0: Diagnostic Interview for ADHD in Adults, second edition (A1: Attention Deficit; A2: hyperactivity/impulsivity); EQ-5D: 5-dimension EuroQol questionnaire; MADRS-S: Montgomery–Åsberg Depression Rating Scale – Self-reported; MINI: Mini International Neuropsychiatric Interview; WAIS-IV: Wechsler Adult Intelligence Scale IV

In the multivariate regression model, a high MADRS-S score (≥ 30) was the strongest predictor of low EQ-5D index scores (adjusted mean, − 0.40; *P* < 0.001, compared with scores between 1 and 11; Fig. 2). MINI-identified anxiety and depression (*P* < 0.001) and having three or more MINI-identified diagnoses (*P* = 0.002) were also strong predictors, compared with having no MINI-identified diagnoses (Fig. 2).

Adjusted mean EQ-5D index scores were significantly higher in men than in women (*P* = 0.049), in patients with secondary education than in those without (*P* = 0.004), and in patients whose main income source was full- or part-time employment, compared with those reliant on other sources (*P =* 0.03, Fig. 2). Adult ASRS-v.1.1 Symptom checklist or DIVA 2.0 scores were not significant predictors of EQ-5D index score (*P ≥* 0.05; Fig. 2).

Almost one quarter (22%) of the patient population had a WAIS-IV score of < 85 but there was no significant association between high WAIS-IV scores and HRQoL.

## Discussion

Adult patients with newly diagnosed ADHD experienced a considerable disease burden and substantially impaired HRQoL in this retrospective chart review. Psychiatric comorbidities were common in the study population, and linear regression analysis identified comorbid anxiety and depression as key factors contributing to poor HRQoL. MADRS-S score was the strongest predictor of EQ-5D index score, implying an association between severe depression and poor HRQoL in adult patients with ADHD. Other variables, including low educational attainment, low levels of employment, and female sex were also associated with poor HRQoL in the study population.

Both the strengths and weaknesses of this study should be considered when interpreting the results. The study included a moderately large number of patients and integrated in-depth analyses of patients’ neuropsychiatric evaluations with a variety of patient- and clinician-reported measures, including HRQoL. The variety of information extracted from the EMRs enabled the investigation of a wide range of potential factors impacting HRQoL in adult patients with ADHD. HRQoL was assessed using the EQ-5D, a validated generic HRQoL tool that provides health utility estimates and is recommended by the UK National Institute for Health and Care Excellence [22]. The use of the generic EQ-5D permits comparisons with reference populations and patients with other diseases [22].

There are, however, several limitations that should be noted. First, data for some parameters were missing for a substantial proportion of patients, which may have introduced bias. To address this, a multiple imputation model was utilized to estimate missing data for the regression model. However, this method may also influence results, particularly when sample sizes are limited. Data were missing for fewer than 20% of the total patients across both sites for all imputed variables except ASRS-v.1.1 score, which was missing for 88% of Liljeholmen patients (Additional file 1: Table S1). This implies that the ASRS-v.1.1 may not be part of the routine diagnostic procedure at Liljeholmen. The high proportion of imputed data for this variable in the linear regression model precludes any reliable conclusions concerning the relationship between ASRS-v.1.1 score and HRQoL in adult patients with ADHD. Secondly, covariates for the statistical models were selected based on the data available, but other factors influencing HRQoL may not have been captured in the study. Thirdly, comorbid psychiatric diagnoses were based on the MINI for all psychiatric disorders except autism. Inclusion of autism as a separate covariate in the analyses may have led to underestimation of the impact of psychiatric comorbidities on patients’ HRQoL. Other limitations to consider are the absence of a comparative control sample of EQ-5D index scores from the Swedish population and the recruitment of patients only in the city of Stockholm.

The findings of the present study strongly suggest that adult patients with ADHD experience reduced HRQoL, and support the conclusion that the manifestations of ADHD are associated with considerable disease burden. The mean EQ-5D index score in the patient population was 0.63 (SD, 0.28), which is markedly lower than published EQ-5D index scores for the healthy adult population in Sweden (range, 0.74–0.89) [23] and in the UK (mean, 0.86; SD, 0.28) [24]. Similarly, low EQ-5D index scores have been reported for patients with other chronic conditions including asthma, chronic obstructive pulmonary disease, diabetes, epilepsy, heart failure, and stroke (overall mean index score, 0.73) [25]. The mean EQ-5D index score in the present study is also within the range reported in a systematic review of HRQoL in adults with psoriasis (0.52–0.9) [26], but higher than that reported for people with spinal complaints (0.39) [27]. The applicability of the present study’s findings to cultural/social groups outside Stockholm is uncertain, although a recent study involving seven countries concluded that the impact of ADHD on patient well-being is consistent across different socioeconomic groups [13]. Furthermore, the EQ-5D scores in the present study are similar to those reported for adult patients with ADHD in other European countries [28, 29], suggesting a broad relevance of the present findings.

To our knowledge, this is the first study to use linear regression to identify specific factors that may influence HRQoL in adult patients with ADHD. Comorbid anxiety and depression, together with patient-rated severe depressive symptoms, were identified as the strongest predictors of poor HRQoL. Given that these diagnoses are frequently comorbid with ADHD, the potential negative impact of these disorders upon patients’ HRQoL should be considered in their clinical management. Adjusted mean EQ-5D index scores were also significantly higher in men than in women, who made up 57% of the patient population. A preponderance of women is uncommon in studies of ADHD, which is diagnosed more frequently in men than in women [2]. Gender is not thought to modulate the phenotypic presentation of ADHD [30], but higher rates of comorbid anxiety disorders have been reported in women than in men [31], which could contribute to the gender difference in HRQoL in the present study.

Psychiatric disorders other than depression and anxiety were common, affecting 19% of the patient population. However, only 3% of the patients in the present study were diagnosed with comorbid bipolar disorder. This is in contrast with the results of the National Comorbidity Study which reported a bipolar disorder prevalence of 19.4% among adults with ADHD [32]. It is possible that the unusually low frequency of bipolar disorder may have influenced the relationship between comorbid psychiatric disorders and HRQoL in the present study, and further studies in patients with comorbid bipolar disorder may be warranted. Over two-thirds of the patient population (77%) also reported at least one somatic comorbidity. Pain was the most frequently reported somatic comorbidity. Individuals with ADHD are more prone to accidents and injuries [33], may be more sensitive to pain [34], and may experience motor inhibition problems and heightened muscle tone [35]. Multiple studies have identified a high incidence of joint hypermobility in individuals with ADHD [36], which may also have contributed to the high prevalence of pain in the present study. Adults with ADHD are more likely to have somatic disorders, and are more likely to use non-psychiatric healthcare services, than those without ADHD [37]. In particular, somatic disorders such as obesity, sleep disorders, and asthma frequently co-occur with ADHD [38]. ADHD symptoms and functional impairment may make people with ADHD more vulnerable to somatic diseases and may affect their ability to obtain appropriate diagnosis and treatment for somatic disease. Optimum care for patients with ADHD may involve an integrated and multidisciplinary approach for identifying and treating co-occurring psychiatric and somatic disorders [39].

Other predictors of poor HRQoL included poor educational achievement and not having a main income derived from employment. In the present study, primary school was the highest level of education reached for 37% of adults with newly diagnosed ADHD, and only 13% were educated to university level. In comparison, equivalent levels in the general Stockholm population in 2014 were reported to be 16 and 45%, respectively [40]. Having an average or higher intelligence score (denoted by a WAIS-IV score of more than 85) was not significantly associated with improved HRQoL, implying that the challenges faced by patients with ADHD in school extend beyond deficits in intellectual functioning. The present results also suggest that difficulties continue into the workplace. Despite a median age of 33 years, fewer than half of the patients were able to support themselves financially through employment or self-employment, and 22% were reliant on sickness benefit as their main source of income. Thus, the present study highlights the significant impact that poor performance in school and the workplace can have on HRQoL and emphasizes the need for early diagnosis and effective clinical management of ADHD symptoms from a young age. The present study was not designed to investigate the impact of ADHD medication on HRQoL in individuals with ADHD, but a recent systematic review concluded that ADHD medication may help to reduce functional impairment and to improve HRQoL deficits [41].

European guidelines recommend an individualized, multimodal approach to treatment of ADHD in adults, including psychological treatments and medication [12]. In the present study, over two-thirds (77%) of patients were prescribed pharmacological treatment for ADHD. This figure is in line with guidance from the Swedish National Board of Health and Welfare indicating that ADHD medication is likely to be effective and appropriate in the majority of patients [42].

## Conclusions

In summary, the findings of this non-interventional, retrospective study indicate that undiagnosed ADHD contributes to poor HRQoL in adults presenting with psychiatric symptoms. Furthermore, the impact of ADHD on HRQoL may be exacerbated by psychiatric comorbidities such as anxiety and depression. Adults with newly diagnosed ADHD and comorbid depression or anxiety may require particular care and resources in their management.

## Additional file

Additional file 1: **Table S1.** Proportion of patients with missing data. (DOCX 16 kb)

## Abbreviations

ADHD: Attention-deficit/hyperactivity disorder
ASRS-v1.1: Adult ADHD Self-Report Scale version 1.1
DIVA 2.0: Diagnostic Interview for ADHD in Adults, second edition (A1: Attention deficit; A2: Hyperactivity/impulsivity)
EQ-5D: 5-dimension EuroQol questionnaire
HRQoL: Health-related quality of life
MADRS-S: Montgomery–Åsberg Depression Rating Scale – Self-reported
MINI: Mini International Neuropsychiatric Interview
PHQ-9: Patient Health Questionnaire 9
SD: Standard deviation
VAS: Visual analogue scale
WAIS-IV: Wechsler Adult Intelligence Scale IV
