# Supplementary material for: Health-related quality of life and burden of illness in adults with newly diagnosed attention-deficit/hyperactivity disorder in Sweden
Source: BMC Psychiatry. 2018 Jul 13;18:223. doi: 10.1186/s12888-018-1803-y (PMC6044069; doi:10.1186/s12888-018-1803-y)
Supplement: Supplementary file 1 — Table S1. Proportion of patients with missing data. (DOCX 16 kb) [file 12888_2018_1803_MOESM1_ESM.docx]

#### Additional file 1: Table S1 Proportion of patients with missing data

| Characteristic | Danderyd  (*n* = 101) | Liljeholmen  (*n* = 88) | Overall  (*N* = 189) |
| --- | --- | --- | --- |
| Patients with missing data, *n* (%) | |  |  |
| ASRS-v.1.1^a^ | 3 (3) | 77 (88) | 80 (42) |
| DIVA 2.0 (A1) | 10 (10) | 16 (18) | 26 (14) |
| DIVA 2.0 (A2) | 15 (15) | 17 (19) | 32 (17) |
| EQ-5D | 6 (6) | 21 (24) | 27 (14) |
| WAIS-IV | 20 (20) | 8 (9) | 28 (15) |
| MADRS-S^b^ | 8 (8) | 16 (18) | 24 (13) |

^a^Symptom checklist

^b^MADRS-S derived from PHQ-9 score for patients enrolled at Liljeholmen (1.206 × PHQ-9) + 4.062

*ADHD* attention-deficit/hyperactivity disorder, *ASRS-v.1.1* Adult ADHD Self-Report Scale version 1.1, *DIVA 2.0* Diagnostic Interview for ADHD in Adults, second edition (A1 Attention deficit, A2 hyperactivity/impulsivity), *EQ-5D* five-dimension EuroQol questionnaire, *MADRS-S* Montgomery–Åsberg Depression Rating Scale – Self-reported, *PHQ-9* Patient Health Questionnaire 9, *WAIS-IV* Wechsler Adult Intelligence Scale
